# Differences in Clinical Characteristics Between Delta Variant and Wild-Type SARS-CoV-2 Infected Patients

**DOI:** 10.3389/fmed.2021.792135

**Published:** 2022-01-03

**Authors:** Zhenkui Hu, Xing Huang, Jianguo Zhang, Shixiang Fu, Daoyin Ding, Zhimin Tao

**Affiliations:** ^1^Department of Critical Care Medicine, The Affiliated Hospital of Jiangsu University, Zhenjiang, China; ^2^Center for Evidence-Based and Translational Medicine, Zhongnan Hospital of Wuhan University, Wuhan, China; ^3^Department of Emergency Medicine, The Affiliated Hospital of Jiangsu University, Zhenjiang, China; ^4^Department of Hepatology, The Third People's Hospital of Yangzhou City, Yangzhou, China; ^5^Department of Critical Care Medicine, The First People's Hospital of Jiangxia District, Wuhan, China; ^6^Jiangsu Province Key Laboratory of Medical Science and Laboratory Medicine, School of Medicine, Jiangsu University, Zhenjiang, China

**Keywords:** SARS-CoV-2, COVID-19, delta variant, vaccination, wild type

## Abstract

**Background:** As delta variant of severe acute respiratory syndrome coronavirus 2 (SARS-CoV-2) prevailed in the current coronavirus disease 2019 (COVID-19) pandemic, its clinical characteristics with the difference from those of wild-type strains have been little studied.

**Methods:** We reported one cohort of 341 wild-type patients with COVID-19 admitted at Wuhan, China in 2020 and the other cohort of 336 delta variant patients with COVID-19 admitted at Yangzhou, China in 2021, with comparisons of their demographic information, medical history, clinical manifestation, and hematological data. Furthermore, within the delta variant cohort, patients with none, partial, and full vaccination were also compared to assess vaccine effectiveness.

**Findings:** For a total of 677 patients with COVID-19 included in this study, their median age was 53.0 years [interquartile range (IQR): 38.0–66.0] and 46.8% were men. No difference was found in age, gender, and percentage of patients with the leading comorbidity between wild-type and delta variant cohorts, but delta variant cohort showed a lessened time interval between disease onset to hospitalization, a reduced portion of patients with smoking history, and a lowered frequency of clinical symptoms. For hematological parameters, most values demonstrated significant differences between wild-type and delta variant cohorts, while full vaccination rather than partial vaccination alleviated the disease condition. This reflected the viremic effect of delta variant when vaccination succeeds or fails to protect.

**Interpretation:** Delta variant of SARS-CoV-2 may cause severe disease profiles, but timely diagnosis and full vaccination could protect patients with COVID-19 from worsened disease progression.

## Introduction

Coronavirus disease 2019 (COVID-19) has been proven to be a highly contagious and fast evolving disease. The responsible pathogen was named severe acute respiratory syndrome coronavirus 2 (SARS-CoV-2), a positive-sense single-stranded RNA virus and the 7th member of the coronavirus family that infects humans (1, 2). Since September 2020, 9 variants of SARS-CoV-2 have been reported and compared to the original strain of the virus with a reproductive number (R_0_) = ~2.5, the recent delta variant showed much higher transmissibility with R_0_ = ~7 (3–6). As of September 7, 2021, the cumulative number of COVID-19 infections has reached over 220 million with a death toll surpassing 4.5 million, while the delta variant has become dominant among the countries with the highest number of newly infected cases including the United States, Brazil, and the United Kingdom (6).

Amid the COVID-19 pandemic, many therapeutics and vaccination strategies have been developed to contain viral spreading. On one hand, repurposed and investigational drugs are heavily studied to reduce viral infection such as (hydroxy) chloroquine, metformin, and remdesivir (7, 8). Among them, orally administered antidepressant fluvoxamine and investigational molnupiravir have shown remarkable inhibition on viral replication, greatly shortening the hospitalization length, and lowering mortality rate (9, 10). On the other hand, a handful of vaccines against COVID-19 that mainly include inactivated vaccines, nucleic acid-based vaccines, and viral vector-based vaccines have been approved for use in different countries (11). As a result, COVID-19 vaccinations effectively lower the transmission of SARS-CoV-2 and decrease the risks of patients for severity and mortality.

Delta variant, or previously known as B.1.617.2, was first reported in India in October 2020 and was imported to China in the middle of May 2021, triggering a new wave of COVID-19 infection across the country (53). The effectiveness of single- or full-dose inactivated vaccines against delta variant infection was 13.8 and 59.0%, respectively, showing decreased protection when compared to that against wild-type SARS-CoV-2 (53). For BNT162b2 (an mRNA vaccine) and ChAdOx1 nCoV-19 (a replication-deficient adenoviral vector vaccine) against COVID-19, their protections from delta variant infection were significantly lower than those from alpha variant infection (12). In parallel, neutralization of delta variant using monoclonal antibody or serum antibody from convalescent patients with COVID-19 demonstrated less sensitivities than that of other SARS-CoV-2 strain (13). On top of that, how the accumulating mutations will affect the antigenicity of SARS-CoV-2 variants remains an imperative puzzle to solve.

Severe acute respiratory syndrome coronavirus 2 employs human angiotensin-converting enzyme 2 (hACE2) for cell entry, infecting lung, heart, and other major organs, and causing hematological disorders and organ impairments (14–16). The virus–host interaction may vary to different extents due to the changing variants of SARS-CoV-2. Simultaneously, since the COVID-19 pandemic began, its clinical characteristics and pathogenic mechanisms have been well-documented (17–20). However, with the rapid spreading of delta variants across the world, their specific clinical features are far from explored. In particular, the difference between characteristics of patients with COVID-19 infected by the wild-type SARS-CoV-2 and its delta variant has yet been elucidated.

In this study, we investigated the clinical features of delta variant infected patients with COVID-19 in Yangzhou, China during August 2021, with a comparison to those of wild-type patients with COVID-19 in Wuhan, China in early 2020. Comparative studies were also conducted to differentiate the unvaccinated patients in Yangzhou with delta variant infection from those in Wuhan with wild-type infection, and from partial (single) or full (two) dose vaccinated patients with COVID-19 in Yangzhou with delta variant infection. Through this study, we aim to understand the unique clinical manifestations of patients with COVID-19 due to infection by delta variant of SARS-CoV-2 and the vaccine efficiency against this delta variant in single or double dosage.

## Methods

### Patients

**Figure 1:**
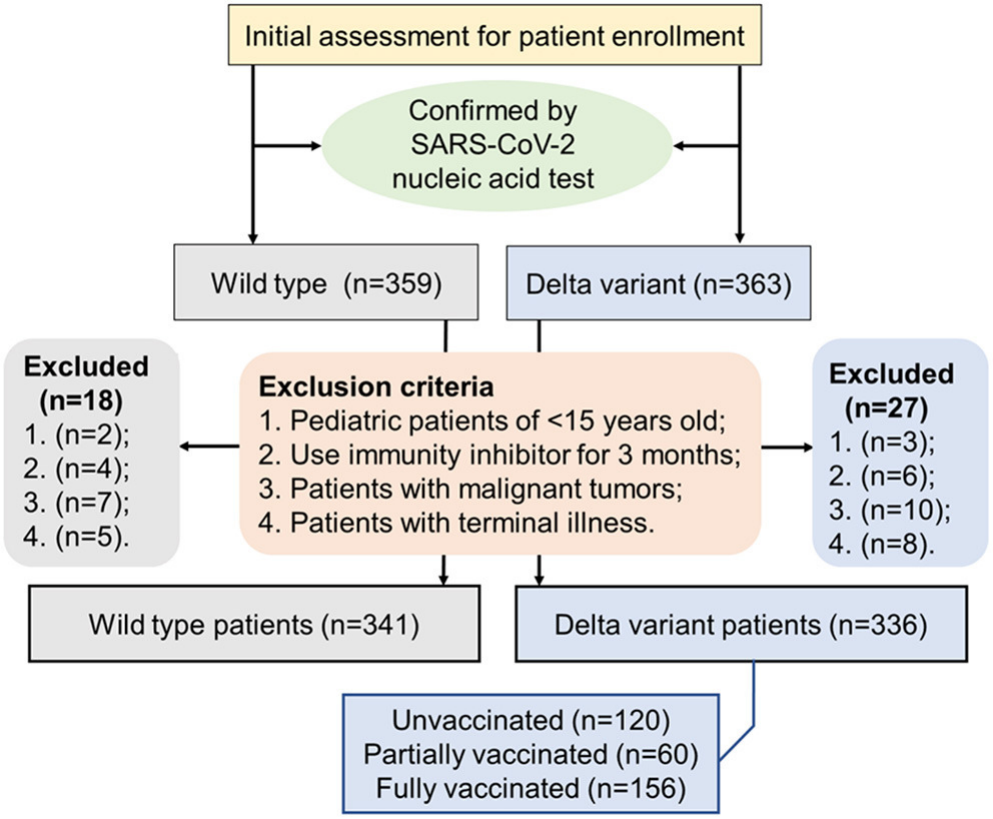
A flowchart displaying the inclusion and exclusion criteria of the patient in the selection procedure of the patient.

This retrospective study included 341 patients with COVID-19 who were admitted and hospitalized at the First People's Hospital of Jiangxia District (FPHJD) in Wuhan City of Hubei Province, China, from January 2020 to April 2020, including 96 patients in the intensive care unit (ICU) and 245 patients in the non-ICU isolation ward. These patients were in the wild-type cohort. In parallel, 336 patients with COVID-19 in the delta variant cohort were admitted and hospitalized at the Third People's Hospital of Yangzhou City (TPHYC), Jiangsu Province, China, in August 2021, where the delta variant of SARS-CoV-2 has been identified as the responsible pathogen (21). No ICU patients were reported in this cohort. For the inclusion criteria, patients with COVID-19 were diagnosed and confirmed by following a standard procedure (22). Exclusion criteria were as follows: (1) pediatric patients of <15 years old; (2) patients that use immunity inhibitor for 3 months and up; (3) patients with malignant tumors; and (4) patients with a terminal illness (Figure 1). This study was approved by the Research Ethics Committee of the FPHJD and the TPHYC, respectively. All the information of the patient remains anonymous and written informed consent was waived due to the emergency of major infectious diseases.

### Vaccinations

For 336 patients with COVID-19 in Yangzhou infected by delta variant of SARS-CoV-2, 120 patients (35.7%) were unvaccinated, 60 patients (17.9%) were partially vaccinated, and 156 patients (46.4%) were fully vaccinated. Two types of inactivated vaccines, Sinovac or Sinopharm (Beijing), were administered. A total of 61 patients (18.2%) were given Sinopharm (Beijing) vaccines, 68 patients (20.2%) were given Sinovac vaccines, and 88 patients (26.2%) were given uncertain vaccines (Sinopharm or Sinovac). A dose of vaccine was counted effective only if the time between the vaccine shot and the disease onset was longer than 14 days. Minimal duration of 14 days was estimated necessary to develop protective immunity against SARS-CoV-2 infection (23). Patients were considered fully vaccinated only if 2 doses of vaccines were given and the time between the 2nd vaccination and the onset of COVID-19 was more than 14 days. Patients were considered partially vaccinated if only 1 effective shot of vaccine was given. Patients were considered unvaccinated if no vaccine had been ever received or the first vaccination was given <14 days before the onset of COVID-19.

### Procedures

Patients with COVID-19 were received and diagnosed by following a standard procedure (24). The wild-type patients were treated with antiviral drugs (oseltamivir, arbidol, and ribavirin), antibiotics (sulperazone, linezolid), antifungal therapy (fluconazole, caspofungin), corticosteroid therapy, respiration-assisted ventilation, and low-molecular-weight heparin (unless an increased risk of bleeding was assessed). For patients with delta variant, they were treated with Chinese traditional medicine (25) and antibiotics (ceftazidime, levofloxacin) if the bacterial infection was assessed. Thymalfasin was subcutaneously injected when the patient suffered from low immune function. All the patients were suggested in prone positions to increase the partial oxygen pressure. Serological tests of patients with COVID-19 based on detection of SARS-CoV-2-specific immunoglobulin M (IgM) and immunoglobulin G (IgG) were conducted, using 2019-nCoV IgG chemiluminescence immunoassay (CLIA) microparticles and 2019-nCoV IgM CLIA microparticles, respectively, manufactured by Autobio Diagnostics Corporation Ltd., China. Blood cell analysis was conducted by automated hematology analyzer (SYSMEX XS or XN series, Japan) and the biochemical indicator was also analyzed (Roche Cobas 8000, USA; Beckman AU5800, USA).

### Statistical Analysis

The categorical variables were described as frequency rates and percentages and continuous variables were applied to describe the median and interquartile range (IQR) values. Comparison of continuous variables between two cohorts was analyzed with the Mann–Whitney U test. Repeated measurements (non-normal distribution) were used following a generalized linear mixed model. The chi-squared test was used to compare the proportion of categorical variables, and Fisher's exact test was employed when data were limited. All the statistical analyses were performed using the GraphPad Prism 5.0 software (GraphPad Software Incorporation, San Diego, California) and statistics analyses adopted published methods (14–16). A two-sided *p* < 0.05 was considered statistically significant.

## Results

A total of 677 patients with COVID-19 were included in this retrospective study, where 341 patients with wild-type infection and 336 patients with delta variant infection were reported from hospitals in Wuhan and Yangzhou, China, respectively. For all the patients in both cohorts, the median age was 53 years (IQR: 38.0–66.0) and 46.8% were men (Table 1). There was no statistical difference in age and gender between wild-type and delta variant SARS-CoV-2-infected patients. However, compared to the wild-type cohort, the delta variant cohort showed a much shorter time duration from disease onset to hospitalization and a much lower portion of patients with smoking history. This corroborates much increased infectivity of the SARS-CoV-2 delta variant, regardless of smoking predisposition. Notably, no ICU patient was admitted and no death case was reported in the delta variant cohort, compared to 28.2% ICU patients and 17.6% non-survival in the wild-type cohort. This result accents the protective role of vaccination against SARS-CoV-2 infection, substantially lessening the severity and mortality of patients with COVID-19.

**Table 1:** Baseline characteristics between COVID-19 patients in wild-type and delta variant cohorts.

	**Total (*n* = 677)**	**Wild type (*n* = 341)**	**Delta variant (*n* = 336)**	***p***
Age	53.0 (38.0–66.0)	54.0 (42.0–66.0)	52.0 (35.0–66.0)	0.057
Gender, male	317 (46.8%)	172 (50.4%)	145 (43.2%)	0.058
Onset to hospitalization, day	3.0 (2.0–5.0)	4.0 (3.0–5.0)	2.0 (1.0–4.0)	<0.0001
Smoking history	122 (18.0%)	92 (27.0%)	30 (8.9%)	<0.0001
Mortality	60 (8.9%)	60 (17.6%)	0 (0)	<0.0001
**Comorbidity**
Hypertension	171 (25.3%)	81 (23.8%)	90 (26.8%)	0.364
Diabetes	73 (10.8%)	42 (12.3%)	31 (9.2%)	0.195
Cardiovascular diseases	36 (5.3%)	14 (4.1%)	22 (6.5%)	0.157
Bronchitis	30 (4.4%)	26 (7.6%)	4 (1.2%)	<0.0001
**Symptoms**
Cough	450 (66.5%)	282 (82.7%)	168 (50.0%)	<0.0001
Fever	397 (58.6%)	276 (80.9%)	121 (36.0%)	<0.0001
Fatigue	206 (30.4%)	130 (38.1%)	76 (22.6%)	<0.0001
Expectoration	97 (14.3%)	58 (17.0%)	39 (11.6%)	0.045
Sore throat	84 (12.4%)	0 (0)	84 (25.0%)	<0.0001
Chest pain	73 (10.8%)	66 (19.4%)	7 (2.1%)	<0.0001
Diarrhea	72 (10.6%)	49 (14.4%)	23 (6.8%)	0.002
Dyspnea	49 (7.2%)	47 (13.8%)	2 (0.6%)	<0.0001
Abdominal pain	49 (7.2%)	45 (13.2%)	4 (1.2%)	<0.0001
Vomiting	39 (5.8%)	37 (10.9%)	2 (0.6%)	<0.0001

For all the patients with COVID-19, hypertension, diabetes, cardiovascular diseases, and bronchitis were the top comorbidities in consistency with our previous findings and others (14, 15, 20, 26). Except for bronchitis, patients infected by wild-type SARS-CoV-2 showed similar frequencies of underlying medical conditions to those infected by delta variant. In parallel, patients with COVID-19 in Yangzhou had a much lowered frequency of bronchitis as a coexisting medical condition, which may be associated with their decreased portion of patients with a smoking history.

Despite delta variant infection, a significantly less portion of patients in Yangzhou showed apparent clinical symptoms compared to that of wild-type patients, except for a new characteristic symptom found common in delta variant-infected patients, i.e., sore throat. For major symptoms, such as cough and fever, patients infected by delta variant SARS-CoV-2 had been found in much lower incidence, indicating weakened viremia or lung infection. In addition, within the delta variant cohort, many COVID-19 symptoms showed marginal occurrence in patients, including dyspnea, chest pain, abdominal pain, diarrhea, and vomiting, suggesting the diminished viremic effect on non-pulmonary organs such as the heart, stomach, and gastrointestinal tract. This again substantiates the various clinical characteristics by viral variant as well as validation of vaccination against SARS-CoV-2 infection.

**Figure 2:**
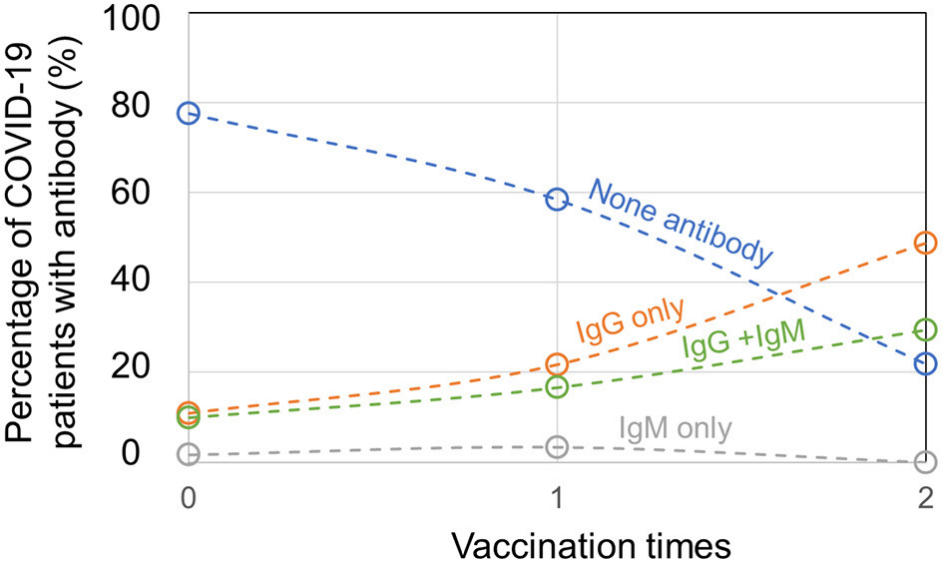
Antibody production in unvaccinated, partially, or fully vaccinated patients with coronavirus disease 2019 (COVID-19).

For 336 patients with COVID-19 infected by delta variant of SARS-CoV-2 in Yangzhou, 120 patients were unvaccinated and among them, 77.5% patients produced no SARS-CoV-2-specific antibody, 10.8% patients produced IgG only, 1.7% patients produced IgM only, and 10.0% patients produced IgG + IgM (Figure 2). The antibody tests of patients with COVID-19 were conducted at their hospital admission. Besides, 60 patients had 1 vaccination, among whom 58.3% patients had no SARS-CoV-2-specific antibody, 21.7% patients had IgG only, 3.3% patients had IgM only, and 16.7% patients had IgG + IgM. For 156 patients who were 2 times vaccinated, 21.8% patients had no SARS-CoV-2-specific antibody, 48.7% patients had IgG only and 29.5% patients had IgG + IgM, while no patient generated IgM only. Importantly, 156 out of 336 confirmed patients with COVID-19 had previously full vaccination, implying a high breakthrough infection rate by delta variant of SARS-CoV-2. Since immunities developed after viral infection or vaccination vary a lot among individuals, and for one individual it is unknown whether SARS-CoV-2 infection and COVID-19 vaccination confer a comparable degree of immunity, positive antibody results from serological tests of vaccinated or unvaccinated patients with COVID-19 may not differentiate their infection or vaccination status. Nevertheless, for confirmed patients with COVID-19, the ratio of patients with no antibody plummeted and the ratio of patients with produced SARS-CoV-2-specific IgG or IgG + IgM climbed when the vaccination times added up.

We further investigated the clinical differences of unvaccinated delta variant patients from wild-type patients with COVID-19 or partially vaccinated or fully vaccinated delta variant patients with COVID-19. Compared to wild-type patients, unvaccinated delta variant patients exhibited similar median age and male ratio, but shorter time duration from disease onset to hospitalization and a lower portion of patients with smoking history (Table 2). Hypertension and diabetes were leading comorbidities in delta variant-infected patients, which remain in similar frequencies to those in patients with wild-type COVID-19. However, clinical symptoms in delta variant patients appeared less occurrent when compared to those of wild-type patients, except for expectoration and sore throat. For baseline blood parameters (Table 3), hematological abnormalities in delta variant patients (unvaccinated) including leukocytosis and neutrophilia were significantly mitigated, but lymphocytopenia was observed similar and monocytosis and thrombocytopenia became much worse compared to those in the wild-type cohort. Furthermore, given derangements in the metabolic proteins and enzymatic biomarkers, levels of C-reactive protein (CRP), alanine aminotransferase (ALT), γ-glutamyl transferase (GGT), lactic dehydrogenase (LDH), and procalcitonin (PCT) were significantly lower in delta variant (unvaccinated) patients than those in wild-type infection, whereas some of the others remained comparable or became much higher such as alkaline phosphatase (ALP), creatinine, and creatine phosphokinase (CPK). For coagulation factors, increased prothrombin time (PT), international normalized ratio (INR), and D-dimer levels were significantly improved in delta variant infection when compared to wild-type COVID-19 infection, whereas prolonged thrombin time and decreased fibrinogen level were worsened. These alterations displayed the differential profiles in clinical characteristics between wild-type and delta variant SARS-CoV-2 infections.

**Table 2:** Comparison of baseline characteristics between delta variant COVID-19 patients and wild-type infection (*) or partially vaccinated delta variant patients (^Δ^) or fully vaccinated delta variant patients (^∥^).

	p*	Wild type (*n* = 341)*	**Delta variant unvaccinated (*n* = 120)**	**Delta variant partially vaccinated (*n* = 60)^Δ^**	***p* ^Δ^**	**Delta variant fully vaccinated (*n* = 156)^**∥**^**	***p* ^∥^**
Age	0.057	54.0 (42.0–66.0)	63.0 (35.3–72.0)	57.5 (42.0–68.8)	0.421	43.0 (33.0-56.8)	<0.0001
Gender, male	0.180	172 (50.4%)	52 (43.3%)	33 (55.0%)	0.139	60 (38.5%)	0.414
Onset to hospitalization, day	<0.0001	4.0 (3.0–5.0)	2.0 (1.0–4.0)	2.0 (1.0–4.0)	0.568	2.0 (1.0-3.0)	0.473
Smoking history	<0.001	92 (27.0%)	13 (10.8%)	3 (5.0%)	0.270	14 (9.0%)	0.606
Mortality	<0.0001	60 (17.6%)	0 (0)	0 (0)	n/a	0 (0)	n/a
**Comorbidity**
Hypertension	0.089	81 (23.8%)	38 (31.7%)	21 (35.0%)	0.653	31 (19.9%)	0.025
Diabetes	0.602	42 (12.3%)	17 (14.2%)	5 (8.3%)	0.338	9 (5.8%)	0.018
Cardiovascular diseases	0.007	14 (4.1%)	13 (10.8%)	4 (6.7%)	0.430	5 (3.2%)	0.014
Bronchitis	0.005	26 (7.6%)	1 (0.8%)	3 (5.0%)	0.109	0 (0)	0.435
**Symptoms**
Cough	<0.0001	282 (82.7%)	64 (53.3%)	28 (46.7%)	0.399	76 (48.7%)	0.447
Fever	<0.0001	276 (80.9%)	44 (36.7%)	23 (38.3%)	0.827	54 (34.6%)	0.724
Fatigue	0.003	130 (38.1%)	28 (23.3%)	18 (30.0%)	0.334	30 (19.2%)	0.407
Expectoration	0.767	58 (17.0%)	19 (15.8%)	7 (11.7%)	0.454	13 (8.3%)	0.054
Sore throat	<0.0001	0 (0)	25 (20.8%)	19 (31.7%)	0.111	40 (25.6%)	0.351
Chest pain	<0.0001	66 (19.4%)	4 (3.3%)	2 (3.3%)	1.000	1 (0.6%)	0.171
Diarrhea	0.028	49 (14.4%)	8 (6.7%)	1 (1.7%)	0.275	14 (9.0%)	0.483
Dyspnea	<0.0001	47 (13.8%)	1 (0.8%)	0 (0)	1.000	1 (0.6%)	1.000
Abdominal pain	<0.0001	45 (13.2%)	1 (0.8%)	0 (0)	1.000	3 (2.0%)	0.635
Vomiting	<0.001	37 (10.9%)	1 (0.8%)	0 (0)	1.000	1 (0.6%)	1.000

**Table 3:** Comparison of blood parameters between delta variant COVID-19 patients and wild-type infection (*) or partially vaccinated delta variant patients (^Δ^) or fully vaccinated delta variant patients (^∥^).

	**Normal range**	p*	Wild type (*n* = 341)*	**Delta variant unvaccinated (*n* = 120)**	**Delta variant partially vaccinated (*n* = 60)^**Δ**^**	***p* ^Δ^**	**Delta variant fully vaccinated (*n* = 156)^**∥**^**	***p* ^∥^**
**Blood cell count**								
White blood cells, × 10^9^/L	3.5–9.5	<0.0001	6.2 (4.7–8.0)	4.8 (3.8–5.7)	5.2 (3.9–6.5)	0.246	5.4 (4.2–6.6)	0.008
Neutrophils, × 10^9^/L	1.8–6.3	<0.0001	4.5 (2.9–6.3)	3.0 (2.1–4.1)	3.2 (2.5–4.7)	0.091	3.5 (2.7–4.7)	0.002
Lymphocytes, × 10^9^/L	1.1–3.2	0.060	1.1 (0.7–1.5)	1.2 (0.9–1.5)	1.0 (0.7–1.4)	0.044	1.1 (0.8–1.5)	0.463
Monocytes, × 10^9^/L	0.1–0.6	<0.001	0.4 (0.3–0.6)	0.5 (0.4–0.7)	0.5 (0.4–0.7)	0.995	0.5 (0.4–0.6)	0.782
Platelet, × 10^9^/L	125–350	<0.0001	193.0 (139.0–261.0)	156.0 (130.0–199.3)	162.0 (108.5–202.0)	0.952	181.5 (140.3–230.5)	<0.001
**Metabolic panel**								
C-reaction protein, mg/L	0–10	<0.0001	25.6 (12.8–64.5)	10.9 (2.5–29.2)	16.3 (6.0–27.7)	0.133	12.3 (3.7–30.5)	0.735
Alanine aminotransferase, U/L	9–50	<0.0001	25.7 (17.8–40.7)	19.5 (12.4–29.7)	17.4 (11.6–27.8)	0.792	17.1 (11.6–30.4)	0.539
Aspartate aminotransferase, U/L	15–40	0.685	26.9 (16.2–46.0)	23.7 (19.1–37.8)	22.7 (18.5–33.0)	0.585	19.9 (15.7–28.0)	<0.001
Alkaline phosphatase, U/L	32–126	<0.0001	70.0 (55.0–97.5)	84.5 (72.0–104.0)	80.5 (65.5–101.5)	0.164	75.0 (65.0–93.0)	0.001
γ-glutamyl transferase, U/L	12–73	<0.0001	48.0 (27.0–74.5)	23.0 (15.0–46.0)	25.5 (16.0–42.0)	0.676	21.0 (14.0–37.0)	0.251
Adenosine deaminase, U/L	0–25	0.222	14.3 (11.2–18.1)	14.0 (12.0–18.0)	14.0 (12.0–17.0)	0.248	13.0 (11.0–15.0)	<0.0001
Blood urea nitrogen, mmol/L	2.86–8.2	0.758	4.7 (3.5–6.2)	4.6 (3.7–5.7)	4.8 (3.7–6.1)	0.834	4.2 (3.3–5.3)	0.004
Creatinine, mmol/L	31.7–133	<0.0001	62.5 (51.2–77.0)	74.0 (62.0–89.5)	72.5 (64.5–88.8)	0.712	67.0 (59.0–81.8)	0.012
**Biomarkers**								
Lactic dehydrogenase, U/L	80–285	<0.0001	373.0 (227.8–542.5)	201.5 (177.0–247.0)	198.5 (180.3–240.5)	0.674	191.0 (166.0–228.5)	0.014
Creatine phosphokinase, U/L	38–174	<0.001	67.0 (47.5–120.5)	94.0 (60.0–146.8)	83.5 (63.0–133.0)	0.915	85.5 (58.0–122.5)	0.393
Creatine kinase isoenzyme, U/L	0–25	<0.0001	47.3 (24.3–72.4)	13.2 (10.6–15.7)	12.5 (9.9–15.6)	0.244	12.5 (10.3–16.9)	0.620
Procalcitonin, ng/mL	<0.1	<0.0001	0.91 (0.38–1.63)	0.04 (0.02–0.05)	0.04 (0.03–0.07)	0.276	0.04 (0.02–0.05)	0.599
**Coagulation factors**								
Prothrombin time, s	9–13	<0.0001	13.6 (12.8–14.3)	12.0 (11.6–12.5)	11.9 (11.4–12.2)	0.147	12.0 (11.4–12.6)	0.751
International normalized ratio	0.8–1.2	<0.001	1.08 (1.01–1.18)	1.05 (1.01–1.09)	1.04 (0.99–1.06)	0.137	1.05 (0.99–1.10)	0.790
Activated partial thromboplastin time, s	23.3–32.5	0.333	30.3 (28.2–32.0)	30.8 (28.0–33.5)	29.9 (27.5–32.5)	0.246	29.9 (26.7–32.5)	0.032
Thrombin time, s	14–21	<0.0001	16.1 (15.1–17.4)	18.2 (17.4–18.9)	17.9 (17.1–18.5)	0.002	17.7 (17.1–18.3)	<0.0001
Fibrinogen, g/L	2–4	<0.001	3.8 (2.7–4.7)	3.2 (2.6–3.9)	3.3 (2.9–4.0)	0.228	3.4 (2.8–4.0)	0.098
D-dimer, mg/L	<0.55	<0.0001	0.74 (0.29–1.92)	0.39 (0.24–0.56)	0.42 (0.24–0.62)	0.914	0.35 (0.21–0.53)	0.216

Furthermore, within the delta variant cohort, partially vaccinated patients showed similar basic characters and clinical profiles to unvaccinated patients, as their baseline data (e.g., median age, male ratio, time between disease onset to hospitalization, and the portion of patients with smoking history), the frequency of patients with major comorbidity, and the commonly observed clinical symptoms owned no statistical difference (Table 2). For hematological parameters, all the tested data showed similar levels, except that lymphocytopenia was slightly aggravated and prolonged thrombin time was alleviated in partially vaccinated patients with COVID-19 (Table 3). These results showed limited protection of single (partial) vaccination from delta variant infection.

In parallel, fully vaccinated patients of delta variant exhibited younger age than unvaccinated patients, although their gender ratio, time from disease onset to hospital admission, the portion of patients with smoking history, and occurrence of clinical manifestations showed no noticeable difference. However, major comorbidities, including hypertension, diabetes, and cardiovascular diseases, demonstrated lower frequencies in patients with full vaccination than those with no vaccination, which might be also associated with their younger ages. Based on laboratory blood tests, many abnormal parameters in fully vaccinated patients showed significant improvements in comparison to those in unvaccinated patients including thrombocytopenia and decreased levels of ALP, adenosine deaminase (ADA), blood urea nitrogen (BUN), creatinine and LDH, and mitigated activated partial thromboplastin time (aPPT) and thrombin time. These results confirmed the validation of full-dose vaccination in preventing disease progression after SARS-CoV-2 delta variant infection.

To conclude, the age, gender, and ratio of patients with the most common comorbidities between wild-type and delta variant cohorts were similar in this study, but the delta variant cohort demonstrated less duration from disease onset to hospitalization, a lower ratio of patients with smoking history, and fewer occurrence of clinical symptoms. This is in corroboration with superior infectivity of the delta variant to the wild type. Many laboratory parameters revealed notable differences between wild-type and delta variant cohorts and within delta variant cohort patients with full vaccination showed much allayed conditions than patients with partial or no vaccination.

## Discussion

Amid the COVID-19 pandemic, the evolution rate of SARS-CoV-2 was estimated to be ~10^−3^ per nucleotide per year or ~3 mutations per month (27, 28). Two specific mutations, G to U and C to U, were found the highest occurrence in SARS-CoV-2, suggesting that the viral genome might be the substrate for certain deaminase or the target for reactive oxygen species in the host (29, 30). Consequently, the delta SARS-CoV-2 possesses a higher viral load, a shorter incubation period, and a longer viral shedding than other known variants or wild-type strains, causing greater transmissibility (31–33).

Enclosing a large single-stranded RNA genome (~30 kb), SARS-CoV-2 virion contains the envelope (E), membrane (M), and spike (S) proteins, where E and M proteins mainly function in the virion assembly and S protein mediates viral entry into the host (34, 35). Structurally, S protein encompasses an N-terminal domain (NTD) harboring a hydrophobic signal sequence motif, an ectodomain including subunit S1 for receptor binding and S2 for membrane fusion, and a transmembrane anchor through virion envelope (36). At the carboxyl end of S1 locates a receptor-binding domain (RBD), in proximity to 2 proteolytic sites to further fuse viral and host cellular membranes: one at the S1/S2 boundary and the other at N-terminus of fusion peptide within S2 (denoted as S2') (37, 38). When SARS-CoV-2 infects, S1 RBD recognizes and binds specific cell receptors (i.e., hACE2), followed by cleavage at the S1/S2 or/and S2' sites by host proteases (e.g., TMPRSS2) to facilitate the viral membrane fusion (39). Thus, antibodies against the S proteins could hamper the viral infection. For this reason, S protein has become the principal target of COVID-19 vaccines, while mutations in S protein may undermine vaccine efficiency (40).

Compared to other SARS-CoV-2 strains, the delta variant possesses notable mutations L452R, T478K, and E484Q in the RBD of S protein and P681R in the S1/S2 site (41). SARS-CoV-2 strains with L452R and E484Q mutations were associated with heightened resistance to antibody neutralization (42). T478K mutation enhanced the infectivity and augmented the ability of SARS-CoV-2 to escape immune recognition (43, 44). Each of the L452R and E484Q mutations lowered susceptibility to mRNA vaccine-generated antibodies by disrupting the binding between the RBD and hACE2, albeit the combined mutations did not show synergism (45). Moreover, within S1/S2 site P681R mutation enabled S protein to be less acidic and made furin in the host much easier to cleave, adding viral infectivity and transmissibility (46). In addition, D614G mutation has been identified in all the variants of concern (VOCs), i.e., alpha, beta, gamma, and delta variants) (47). This replacement is associated with heightened viral load and lowered the age of the patient, but it has no effect on the disease severity and mortality of COVID-19 (48). Taken together, those accumulating mutations in delta variants contribute to their increased virulence and transmissibility and decreased antibody neutralization and vaccine efficiency.

Recently, in a highly vaccinated health system in California, USA, 57.3% of medical workers who were infected with delta variant of SARS-CoV-2 had full mRNA vaccination record, being a result of both the viral mutation and declined immunity over time (49). It was noted in this study that 64.3% of patients with partial or full vaccination were infected by delta variant of SARS-CoV-2, albeit 46.4% patients with full vaccination had younger median age and lower occurrence of the most common comorbidities associated with COVID-19 infection (14, 15). This again corroborates the elevated infectivity of the SARS-CoV-2 delta variant and its augmented capacity of the breakthrough of the vaccine.

This study indicated that delta variant infection caused less symptom occurrence than wild-type infection. Cough and fever are still predominant signs, but gastrointestinal symptoms have become much less frequent. This result is consistent with other findings (33). Additionally, sore throat that was not common in patients with wild-type COVID-19 has been found in a substantial portion of delta variant patients. For hematological data, in comparison to wild-type infections, delta variant patients (unvaccinated) demonstrated alleviated leukocytosis, neutrophilia, levels of CRP, ALT, GGT, LDH, PCT, PTT, INR, and D-dimer, but deteriorated monocytosis, thrombocytopenia, levels of ALP, creatinine, CPK, thrombin time, and fibrinogen, showing a varying set of clinical characteristics. Partial vaccination did not substantially alleviate the severity of delta variant infection, while full vaccination significantly ameliorated the condition of the patients by amending coagulation dysfunction (including thrombocytopenia, prolonged aPPT, and thrombin time) and extenuating viremic impact on major organs (typified by reduced levels of ALP, ADA, BUN, creatinine, and LDH). These results pointed to that despite lowered effectiveness against delta variant, COVID-19 vaccination results in significantly reduced hospitalization and disease progression. This finding echoes with other studies (50, 51).

This study contains several limitations. First, compared to that in Wuhan, the local outbreak in Yangzhou with delta variant of SARS-CoV-2 adopted a different containment policy, where a city-wide nucleic acid testing was conducted immediately after the first few patients were identified. As a result, positive patients with COVID-19 were quickly quarantined and hospitalized for treatment, largely preventing the disease from worsening (52). Second, the size of the study cohort is low, especially for the delta variant cohort that was further divided into unvaccinated, partially vaccinated, and fully vaccinated. Comparison between small-size groups may not convey the most representative results. Third, the vaccine efficiency may not be assessed in this retrospective study, as all the cases included are confirmed patients with COVID-19 and acquired immunity against SARS-CoV-2 due to infection or vaccination cannot be distinguished, as it continues to decline along with changing mutations of the virus.

In this study, we reported the clinical profile of delta variant-infected patients with COVID-19 differing from wild-type infection and demonstrated the effective protection of inactivated vaccines against delta variant of SARS-CoV-2, although the vaccine designs were originally based on wild-type viral strains. Two solid conclusions can be made through this study: first, mass nucleic acid testing strategies can diagnose and, therefore, treat patients with COVID-19 in a timely manner, effectively confining the virus spreading and shortening the time interval between disease onset to hospitalization; second, sufficient vaccination can protect patients with COVID-19, once infected, from further disease progression into severity, greatly neutralizing the viremic effect in a preventive manner. Together, stringent adoption of these strategies could help to banish the COVID-19 pandemic soon.

## Data Availability Statement

The datasets presented in this article are not readily available: data are available on request due to privacy/ethical restrictions.

## Ethics Statement

The studies involving human participants were reviewed and approved by research Ethics Committees of the First People's Hospital of Jiangxia District at Wuhan and the Third People's Hospital of Yangzhou City. The ethics committee waived the requirement of written informed consent for participation.

## Author Contributions

JZ and ZT conceived the idea and designed the study. ZH, JZ, SF, DD, and ZT had approved access to all the data in the study. XH and ZT contributed to the statistical analysis. All authors contributed to data analysis, manuscript writing, reviewed, and approved the submission of the manuscript.

## Conflict of Interest

The authors declare that the research was conducted in the absence of any commercial or financial relationships that could be construed as a potential conflict of interest.

## Publisher's Note

All claims expressed in this article are solely those of the authors and do not necessarily represent those of their affiliated organizations, or those of the publisher, the editors and the reviewers. Any product that may be evaluated in this article, or claim that may be made by its manufacturer, is not guaranteed or endorsed by the publisher.

## References

[1] Wu F Zhao S Yu B Chen YM Wang W Song ZG. A new coronavirus associated with human respiratory disease in China. Nature. (2020) 579:265–9. 10.1038/s41586-020-2008-3 32015508 PMC7094943

[2] Zhou F Yu T Du R Fan G Liu Y Liu Z. Clinical course and risk factors for mortality of adult inpatients with COVID-19 in Wuhan, China: a retrospective cohort study. Lancet. (2020) 395:1054–62. 10.1016/S0140-6736(20)30566-3 32171076 PMC7270627

[3] Anand U Cabreros C Mal J Ballesteros F Jr Sillanpaa M Tripathi V. Novel coronavirus disease 2019 (COVID-19) pandemic: from transmission to control with an interdisciplinary vision. Environ Res. (2021) 197:111126. 10.1016/j.envres.2021.111126 33831411 PMC8020611

[4] Burki TK. Lifting of COVID-19 restrictions in the UK and the Delta variant. Lancet Respir Med. (2021) 9:e85. 10.1016/S2213-2600(21)00328-3 34265238 PMC8275031

[5] Mahase E. Covid-19: how many variants are there, and what do we know about them? BMJ. (2021) 374:n1971. 10.1136/bmj.n1971 34413059

[6] Organization WH. (2021). COVID-19 weekly epidemiological update, 2 *March* 2021.

[7] Sanders JM Monogue ML Jodlowski TZ Cutrell JB. Pharmacologic treatments for Coronavirus Disease 2019 (COVID-19): a review. JAMA. (2020) 323:1824–36. 10.1001/jama.2020.6019 32282022

[8] Shoshan-Barmatz V Anand U Nahon-Crystal E Di Carlo M Shteinfer-Kuzmine A. Adverse effects of metformin from diabetes to COVID-19, Cancer, Neurodegenerative Diseases, and Aging: is VDAC1 a common target? Front Physiol. (2021) 12:730048. 10.3389/fphys.2021.730048 34671273 PMC8521008

[9] Lenze EJ Mattar C Zorumski CF Stevens A Schweiger J Nicol GE. Fluvoxamine vs Placebo and clinical deterioration in outpatients with symptomatic COVID-19: a randomized clinical trial. JAMA. (2020) 324:2292–300. 10.1001/jama.2020.22760 33180097 PMC7662481

[10] Mahase E. Covid-19: Molnupiravir reduces risk of hospital admission or death by 50% in patients at risk, MSD reports. BMJ. (2021) 375:n2422. 10.1136/bmj.n2422 34607801

[11] Anand U Jakhmola S Indari O Jha HC Chen ZS Tripathi V. Potential therapeutic targets and vaccine development for SARS-CoV-2/COVID-19 pandemic management: a review on the recent update. Front Immunol. (2021) 12:658519. 10.3389/fimmu.2021.658519 34276652 PMC8278575

[12] Lopez Bernal J Andrews N Gower C Gallagher E Simmons R Thelwall S. Effectiveness of Covid-19 vaccines against the B.1.617.2 (Delta) variant. N Engl J Med. (2021) 385:585–94. 10.1056/NEJMoa2108891 34289274 PMC8314739

[13] Planas D Veyer D Baidaliuk A Staropoli I Guivel-Benhassine F Rajah MM. Reduced sensitivity of SARS-CoV-2 variant Delta to antibody neutralization. Nature. (2021) 596:276–80. 10.1038/s41586-021-03777-9 34237773

[14] Zhang J Ding D Huang X Zhang J Chen D Fu P. Differentiation of COVID-19 from seasonal influenza: a multicenter comparative study. J Med Virol. (2021) 93:1512–9. 10.1002/jmv.26469 32856744 PMC7461066

[15] Zhang J Huang X Ding D Tao Z. Platelet-driven coagulopathy in COVID-19 patients: in comparison to seasonal influenza cases. Exp Hematol Oncol. (2021) 10:34. 10.1186/s40164-021-00228-z 34059141 PMC8165133

[16] Zhang J Huang X Ding D Zhang J Xu L Hu Z. Comparative study of acute lung injury in COVID-19 and Non-COVID-19 patients. Front Med. (2021) 8:666629. 10.3389/fmed.2021.666629 34485324 PMC8415545

[17] Guan WJ Ni ZY Hu Y Liang WH Ou CQ He JX. China medical treatment expert group for, clinical characteristics of Coronavirus Disease 2019 in China. N Engl J Med. (2020) 382:1708–20. 10.1056/NEJMoa2002032 32109013 PMC7092819

[18] Wang D Hu B Hu C Zhu F Liu X Zhang J. Clinical characteristics of 138 hospitalized patients with 2019 novel coronavirus-infected pneumonia in Wuhan, China. JAMA. (2020) 323:1061–9. 10.1001/jama.2020.1585 32031570 PMC7042881

[19] Yang X Yu Y Xu J Shu H Xia J Liu H. Clinical course and outcomes of critically ill patients with SARS-CoV-2 pneumonia in Wuhan, China: a single-centered, retrospective, observational study. Lancet Respir Med. (2020) 8:475–81. 10.1016/S2213-2600(20)30079-5 32105632 PMC7102538

[20] Zhou P Yang XL Wang XG Hu B Zhang L Zhang W. A pneumonia outbreak associated with a new coronavirus of probable bat origin. Nature. (2020) 579:270–3. 10.1038/s41586-020-2012-7 32015507 PMC7095418

[21] Zhou L Nie K Zhao H Zhao X Ye B Wang J. Eleven COVID-19 outbreaks with local transmissions caused by the imported SARS-CoV-2 Delta VOC—China, July–August, 2021. China CDC Wkly. (2021) 3:863–8. 10.46234/ccdcw2021.213 34703643 PMC8521157

[22] Huang C Wang Y Li X Ren L Zhao J Hu Y. Clinical features of patients infected with 2019 novel coronavirus in Wuhan, China. Lancet. (2020) 395:497–506. 10.1016/S0140-6736(20)30183-5 31986264 PMC7159299

[23] Zhang Y Zeng G Pan H Li C Hu Y Chu K. Safety, tolerability, and immunogenicity of an inactivated SARS-CoV-2 vaccine in healthy adults aged 18-59 years: a randomised, double-blind, placebo-controlled, phase 1/2 clinical trial. Lancet Infect Dis. (2021) 21:181–92. 10.1016/S1473-3099(20)30843-4 33217362 PMC7832443

[24] Li T. Diagnosis and clinical management of severe acute respiratory syndrome Coronavirus 2 (SARS-CoV-2) infection: an operational recommendation of Peking Union Medical College Hospital (V2.0). Emerg Microbes Infect. (2020) 9:582–5. 10.1080/22221751.2020.1735265 32172669 PMC7103730

[25] Ren JL Zhang AH Wang XJ. Traditional Chinese medicine for COVID-19 treatment. Pharmacol Res. (2020) 155:104743. 10.1016/j.phrs.2020.104743 32145402 PMC7128263

[26] Shi H Han X Jiang N Cao Y Alwalid O Gu J. Radiological findings from 81 patients with COVID-19 pneumonia in Wuhan, China: a descriptive study. Lancet Infect Dis. (2020) 20:425–34. 10.1016/S1473-3099(20)30086-4 32105637 PMC7159053

[27] Duchene S Featherstone L Haritopoulou-Sinanidou M Rambaut A Lemey P Baele G. Temporal signal and the phylodynamic threshold of SARS-CoV-2. Virus Evol. (2020) 6:veaa061. 10.1093/ve/veaa061 33235813 PMC7454936

[28] Sender R Bar-On YM Gleizer S Bernshtein B Flamholz A Phillips R. The total number and mass of SARS-CoV-2 virions. Proc Natl Acad Sci USA. (2021) 118:e2024815118. 10.1073/pnas.2024815118 34083352 PMC8237675

[29] De Maio N Walker CR Turakhia Y Lanfear R Corbett-Detig R Goldman N. Mutation rates and selection on synonymous mutations in SARS-CoV-2. Genome Biol Evol. (2021) 13:evab087. 10.1093/gbe/evab087 33895815 PMC8135539

[30] Mourier T Sadykov M Carr MJ Gonzalez G Hall WW Pain A. Host-directed editing of the SARS-CoV-2 genome. Biochem Biophys Res Commun. (2021) 538:35–9. 10.1016/j.bbrc.2020.10.092 33234239 PMC7643664

[31] Ong SWX Chiew CJ Ang LW Mak TM Cui L Toh MPHS. Clinical and virological features of SARS-CoV-2 variants of concern: a retrospective cohort study comparing B.1.1.7 (Alpha), B.1.315 (Beta), and B.1.617.2 (Delta). Clin Infect Dis. (2021) ciab721. 10.2139/ssrn.3861566 34423834 PMC8522361

[32] Teyssou E Delagrèverie H Visseaux B Lambert-Niclot S Brichler S Ferre V. The Delta SARS-CoV-2 variant has a higher viral load than the Beta and the historical variants in nasopharyngeal samples from newly diagnosed COVID-19. J Infect. (2021) 83:e1–e3. 10.1016/j.jinf.2021.08.027 34419559 PMC8375250

[33] Wang R Chen J Gao K Wei GW. Vaccine-escape and fast-growing mutations in the United Kingdom, the United States, Singapore, Spain, India, and other COVID-19-devastated countries. Genomics. (2021) 113:2158–70. 10.1016/j.ygeno.2021.05.006 34004284 PMC8123493

[34] Letko M Marzi A Munster V. Functional assessment of cell entry and receptor usage for SARS-CoV-2 and other lineage B betacoronaviruses. Nat Microbiol. (2020) 5:562–9. 10.1038/s41564-020-0688-y 32094589 PMC7095430

[35] Finkel Y Mizrahi O Nachshon A Weingarten-Gabbay S Morgenstern D Yahalom-Ronen Y. The coding capacity of SARS-CoV-2. Nature. (2021) 589:125–30. 10.1038/s41586-020-2739-1 32906143

[36] Li F. Structure, function, and evolution of coronavirus spike proteins. Ann Rev Virol. (2016) 3:237–61. 10.1146/annurev-virology-110615-042301 27578435 PMC5457962

[37] Coutard B Valle C de Lamballerie X Canard B Seidah NG Decroly E. The spike glycoprotein of the new coronavirus 2019-nCoV contains a furin-like cleavage site absent in CoV of the same clade. Antiviral Res. (2020) 176:104742. 10.1016/j.antiviral.2020.104742 32057769 PMC7114094

[38] Walls AC Park YJ Tortorici MA Wall A McGuire AT Veesler D. Structure, function, and antigenicity of the SARS-CoV-2 spike glycoprotein. Cell. (2020) 181:281–92 e6. 10.1016/j.cell.2020.02.058 32155444 PMC7102599

[39] Hoffmann M Kleine-Weber H Schroeder S Kruger N Herrler T Erichsen S. SARS-CoV-2 cell entry depends on ACE2 and TMPRSS2 and is blocked by a clinically proven protease inhibitor. Cell. (2020) 181:271–80 e8. 10.1016/j.cell.2020.02.052 32142651 PMC7102627

[40] Martinez-Flores D Zepeda-Cervantes J Cruz-Resendiz A Aguirre-Sampieri S Sampieri A Vaca L. SARS-CoV-2 vaccines based on the spike glycoprotein and implications of new viral variants. Front Immunol. (2021) 12:701501. 10.3389/fimmu.2021.701501 34322129 PMC8311925

[41] Khateeb J Li Y Zhang H. Emerging SARS-CoV-2 variants of concern and potential intervention approaches. Crit Care. (2021) 25:244. 10.1186/s13054-021-03662-x 34253247 PMC8274962

[42] Verghese M Jiang B Iwai N Mar M Sahoo MK Yamamoto F. A SARS-CoV-2 variant with L452R and E484Q neutralization resistance mutations. J Clin Microbiol. (2021) 59:e0074121. 10.1128/JCM.00741-21 33952596 PMC8218743

[43] Di Giacomo S Mercatelli D Rakhimov A Giorgi FM. Preliminary report on severe acute respiratory syndrome coronavirus 2 (SARS-CoV-2) spike mutation T478K. J Med Virol. (2021) 93:5638–43. 10.1002/jmv.27062 33951211 PMC8242375

[44] Wang Y Chen R Hu F Lan Y Yang Z Zhan C. Transmission, viral kinetics and clinical characteristics of the emergent SARS-CoV-2 Delta VOC in Guangzhou, China. EClinicalMedicine. (2021) 40:101129. 10.1016/j.eclinm.2021.101129 34541481 PMC8435265

[45] Ferreira I Kemp S Datir R Saito A Meng B Rakshit P. SARS-CoV-2 B. 1.617 mutations L452 and E484Q are not synergistic for antibody evasion. J Infect Dis. (2021) 224:989–94. 10.1093/infdis/jiab368 34260717 PMC8420622

[46] Callaway E. The mutation that helps Delta spread like wildfire. Nature. (2021) 596:472–3. 10.1038/d41586-021-02275-2 34417582

[47] Konings F Perkins MD Kuhn JH Pallen MJ Alm EJ Archer BN. SARS-CoV-2 Variants of interest and concern naming scheme conducive for global discourse. Nat Microbiol. (2021) 6:821–3. 10.1038/s41564-021-00932-w 34108654

[48] Volz E Hill V McCrone JT Price A Jorgensen D O'Toole A. Evaluating the effects of SARS-CoV-2 Spike mutation D614G on transmissibility and pathogenicity. Cell. (2021) 184:64–75 e11. 10.1016/j.cell.2020.11.020 33275900 PMC7674007

[49] Keehner J Horton LE Binkin NJ Laurent LC Pride D Longhurst CA. Resurgence of SARS-CoV-2 infection in a highly vaccinated health system workforce. N Engl J Med. (2021) 385:1330–2. 10.1056/NEJMc2112981 34469645 PMC8451183

[50] Antonelli M Penfold RS Merino J Sudre CH Molteni E Berry S. Risk factors and disease profile of post-vaccination SARS-CoV-2 infection in UK users of the COVID Symptom Study app: a prospective, community-based, nested, case-control study. Lancet Infect Dis. (2021) S1473-3099(21)00460-6. 10.1016/S1473-3099(21)00460-6 34480857 PMC8409907

[51] Thangaraj JWV Yadav P Kumar CG Shete A Nyayanit DA Rani DS. Predominance of delta variant among the COVID-19 vaccinated and unvaccinated individuals, India, May 2021. J Infect. (2021) S0163-4453(21)00387-X. 10.1016/j.jinf.2021.08.006 34364949 PMC8343391

[52] Li Z Liu F Cui J Peng Z Chang Z Lai S. Comprehensive large-scale nucleic acid-testing strategies support China's sustained containment of COVID-19. Nat Med. (2021) 27:740–2. 10.1038/s41591-021-01308-7 33859409

[53] Li XN Huang Y Wang W Jing QL Zhang CH Qin PZ. Efficacy of inactivated SARS-CoV-2 vaccines against the Delta variant infection in Guangzhou: a test-negative case-control real-world study. Emerg Microbes Infect. (2021) 10:1751–9. 10.1080/22221751.2021.1969291 34396940 PMC8425710

